# Euler diagrams drawn with ellipses area-proportionally (Edeap)

**DOI:** 10.1186/s12859-021-04121-8

**Published:** 2021-04-26

**Authors:** Michael Wybrow, Peter Rodgers, Fadi K. Dib

**Affiliations:** 1 Faculty of Information Technology, Monash University, 3800 Clayton, Australia; 2 School of Computing, University of Kent, CT2 7NZ Canterbury, UK; 3 Computer Science Department, Gulf University for Science and Technology, 32093 Hawally, Kuwait

**Keywords:** Euler diagrams, Diagram generation, Ellipses, Area proportional, Multi-criteria optimization

## Abstract

**Background:**

Area-proportional Euler diagrams are frequently used to visualize data from Microarray experiments, but are also applied to a wide variety of other data from biosciences, social networks and other domains.

**Results:**

This paper details Edeap, a new simple, scalable method for drawing area-proportional Euler diagrams with ellipses. We use a search-based technique optimizing a multi-criteria objective function that includes measures for both area accuracy and usability, and which can be extended to further user-defined criteria. The Edeap software is available for use on the web, and the code is open source. In addition to describing our system, we present the first extensive evaluation of software for producing area-proportional Euler diagrams, comparing Edeap to the current state-of-the-art; circle-based method, venneuler, and an alternative ellipse-based method, eulerr.

**Conclusions:**

Our evaluation—using data from the Gene Ontology database via GoMiner, Twitter data from the SNAP database, and randomly generated data sets—shows an ordering for accuracy (from best to worst) of eulerr, followed by Edeap and then venneuler. In terms of runtime, the results are reversed with venneuler being the fastest, followed by Edeap and finally eulerr. Regarding scalability, eulerr cannot draw non-trivial diagrams beyond 11 sets, whereas no such limitation is present in Edeap or venneuler, both of which draw diagrams up to the tested limit of 20 sets.

## Background

The motivation behind this work is the demand for area-proportional Euler diagrams to visualize data in areas such as biosciences, social networks and numerous other disciplines [1]. In particular, such diagrams are frequently used to visualize Microarray experiments [2], where diagrams can show what flagged genes are shared by different categories, perhaps revealing insights such as unexpected similarities between different categories.

Set cardinality visualization using Euler diagrams attempts to draw set intersections, represented by interlinking closed curves, area-proportionally, so that the areas of overlapping regions are directly proportional to the cardinality in the input data. Hence, the regions formed from curve overlaps should have area proportions based on the input data, or *area specification*. The area specification gives the required set intersections and their cardinality information.

Until recently, users had a choice between reasonably accurate ellipse-based diagrams limited to three sets [3], or the typically inaccurate diagrams drawn with circle-based methods [4], which allow a larger number of sets.

Tools that use 3 circles forming Venn-3 are known to be inaccurate [5]. Inaccuracies worsen as the number of sets increases. Fortunately, it is known that far more accurate diagrams can be drawn when ellipses are used in place of circles, with accuracy rates well over 90% achievable for Venn-3 diagrams [3]. Prior to the systems discussed in this paper, no method has been developed to draw diagrams beyond three sets with ellipses.

The earliest area-proportional work considered Venn-2 drawn as two circles [6], which can always be drawn exactly no matter the cardinality of the set intersections. However the subsequent work on circle-based Venn-3 diagrams [7, 8], demonstrated the inaccuracy inherent in this more complex type of diagram. This is because three circles do not have enough degrees of freedom. For example, although it is possible to obtain the exact overlapping areas of the paired circles, this means that the shape and size of the middle region overlapping three circles is fixed and therefore probably does not correspond to the required cardinality for this region.

Systems drawing an arbitrary number of sets with circles began with VennMaster [2], followed by venneuler [4]. Whilst it is acknowledged that, in general, as scale increases, visualizing cardinality information with circles becomes highly inaccurate [9], venneuler was shown to be more accurate, at least in terms of the *stress* measure that venneuler uses in its optimizer. Stress measures normalized loss using sums of squared residuals (i.e., the sum of squared differences between the desired area of each disjoint region and the actual count of elements in that region) divided by the total sums of squares (i.e., sums of actual count of elements in each disjoint region). In an attempt to improve accuracy beyond what is possible with circles, the EulerApe system was developed which draws Venn-3 diagrams with ellipses [3]. EulerApe achieved a 98% success rate in accurately drawing randomly generated area specifications against a 0% success for circle-based Venn-3 diagrams.

Preliminary work using rectilinear shapes, that is polygons formed of line segments intersecting at right angles, have been explored [10]. However, rectangles and squares have been shown to be poor choices for Euler diagram comprehension [11], and so we believe diagrams using more complex rectilinear shapes would be even worse for usability.

**Table 1:** A comparison of features of Edeap with other popular tools used to generate Venn Diagrams

Category	VennDiagram Web [12]	BioVenn [8]	GeneVenn [13]	vennMaster [2]	venneuler [4]	eulerr [14]	Edeap
Shapes	Ellipses	Circles	Circles	Circles	Circles	Ellipses	Ellipses
Max no. of sets	5	3	3	> 11	> 11	11	> 11
Move/change shapes	No	No	No	Yes	Yes	Yes	Yes
Area proportional	No	Yes	No	Yes	Yes	Yes	Yes

Hence, the low comprehension associated with rectilinear diagrams and the high levels of inaccuracy present in circle visualizations call for an ellipse solution to the general case. VennDiagram [15], and later VennDiagramWeb [12] is a diagrammatic package that uses ellipses for up to 5 set diagrams. However, this software is restricted to a few predefined diagram layouts. BioVenn [8] and GeneVenn [13] have similar restrictions. VennMaster [2] has no limit on the number of circles it can visualize, but a study provided evidence that venneuler had improved performance [4]. Table 1 presents a comparison of features of Edeap to other popular tools used to generate Venn/Euler diagrams. The comparison criteria include: the shapes drawn by the tools; the maximum number of sets that can be drawn; fixed or movable shapes (fixed means the shape position is not moved automatically); and area proportionality (whether the diagrams are drawn with an attempt at matching region areas to input data).

The eulerr system [14, 16], is a method developed simultaneously, but entirely independently of our Edeap system. It shares the same approach of drawing arbitrary sets with ellipses. This work relies on the stress measure from venneuler as a target function. The initial layout uses only circles and applies multidimensional scaling on the pairwise comparison of circle overlap areas. The optimizer first attempts the use of the nlm (non-linear minimization) optimizer from the R stats package to minimize stress, using 5 variables from each ellipse: x,y coordinates, rotation and relationship between the semiaxes. If the diagram is not accurate after this first search, a last-ditch optimizer is applied, using generalized simulated annealing from the R package GenSA, with the same variables and target function. This last method is considerably more computationally expensive than nlm.

Non-Euler approaches to visualizing set cardinality information have been proposed, including: Fan Diagrams [17]; network diagrams [18]; and the hybrid technique UpSet which claims to be able to visualize up to 20–30 sets [19]. However, none of these use the intuitive intersecting smooth curves that are familiar to users. Using any of these methods requires training, whereas a user can apply area-proportional Euler diagrams to visualize their data without needing to explain the visualization technique.

Our research goals were to increase the number of sets that can be drawn with Euler diagrams that use ellipses, and to compare the capabilities of existing systems. As a result, we developed Edeap, a web-based software system that can accurately draw diagrams representing a larger number of sets compared to previous techniques, matching the scale of data that UpSet can visualize.

**Fig. 1:**
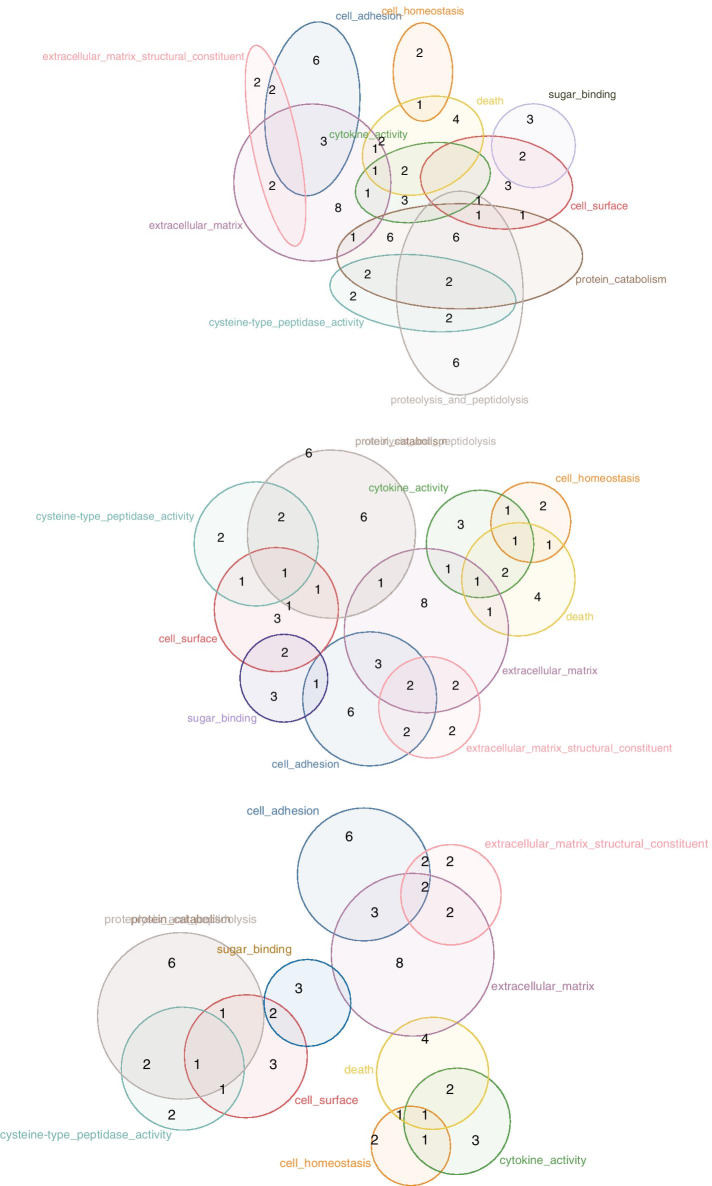
The same GO Miner data visualized with: our approach (Edeap), top; eulerr, middle; and venneuler, bottom. Edeap label placement and drawing is used in all cases. GO Miner filters, p-value: 0.05, minimum: 35, maximum 160

As venneuler is considered the most accurate circle-based method, and eulerr takes a similar approach as Edeap, we chose to compare these three methods for accuracy, scalability and time performance. Figure 1 shows an example of the same data set drawn with the three systems.

## Implementation

Edeap is a web-based system for drawing area-proportional Euler diagrams using ellipses. It is open source and available to use at [20]. We designed Edeap to achieve a combination of scalabilty and accuracy. The system is implemented as a web page allowing it to be easily used by a wide audience. Since it uses multi-criteria optimisation, Edeap is readily extensible with further user-defined criteria for aesthetic and domain specific requirements.

We measure a number of criteria in the diagram. This includes the statistical accuracy of the diagram, but also includes features such as the closeness of ellipses to each other, missing regions and extra (unwanted) regions. These are then placed in a weighted sum. The appropriate weights for each criteria were derived through experimentation. We then compared a hill climbing search against a simulated annealing method. After experimentation we chose the hill climbing method, as it was much faster without any noticeable reduction in accuracy. The weights for Edeap were then tuned on a randomly generated data set.

The Edeap tool allows a user to enter a textual area specification, click a button and get back the resulting diagram. The input to Edeap is a text description of the desired set intersections and the cardinality of each intersection. In the drawn diagram, each set will be drawn with an ellipse, and we will attempt to represent each intersection with a region bounded by only the ellipses corresponding to the sets given in the intersection. Moreover, to achieve area-proportionality we aim to make each region area proportional to the intersection cardinality.

**Fig. 2:**
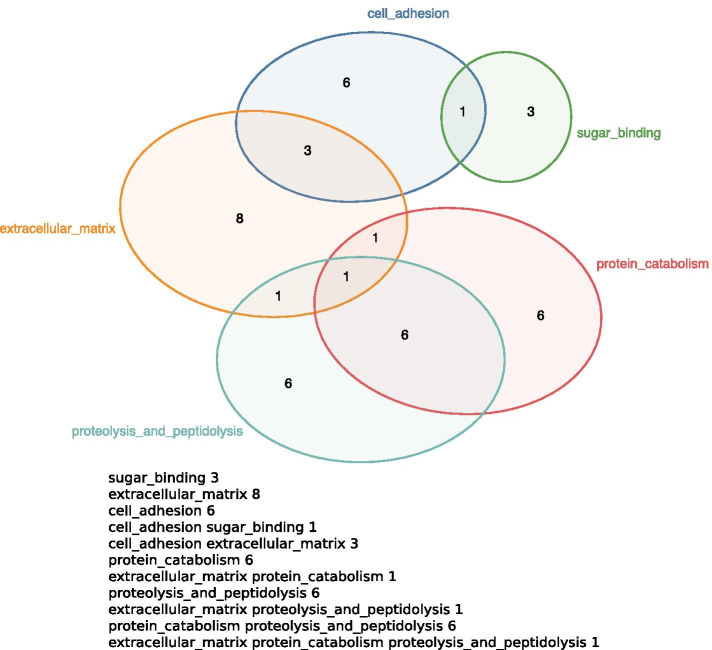
An Edeap diagram (top) accurately representing the given area specification (bottom)

Figure 2 shows an example area specification and resultant diagram. The labels identify the intended sets (e.g., “cell_adhesion” or “sugar_binding”). In the resultant diagram, an ellipse will appear for each distinct set label. We can describe the set intersections, and so the number of ellipses that should overlap in a region by the number of labels in the specification. E.g., the line, “cell_adhesion extracellular_matrix 3” should result in a two-label region of size 3.

Edeap produces images in SVG format, which can be converted to common formats like GIF, TIFF and PNG using freely available SVG editors such as Inkscape [21]. Whilst our intention is to provide a basic layout tool with output that can be customized by third party editors, Edeap has options allowing the user to control the diagram size, colour palette and label sizes for set labels and specified region area values (including hiding them completely). Edeap is implemented in JavaScript and has no external dependencies.

**Fig. 3:**
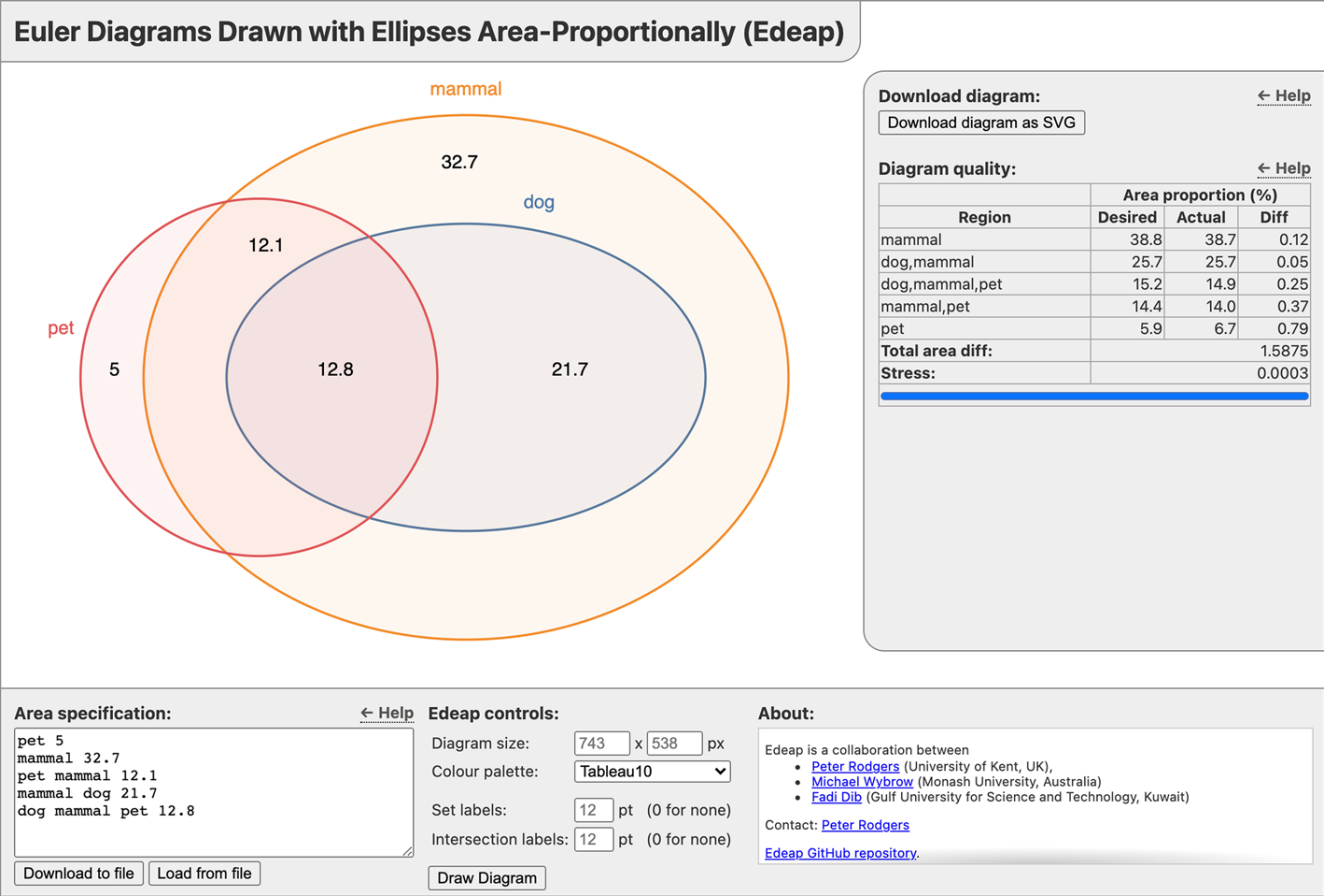
Screenshot of the Edeap web-based diagram generation tool, available at https://www.eulerdiagrams.com/edeap/. The generated diagram is shown in the main part of the view. The right-hand section shows information on the accuracy of the generated diagram and allows the user to download the diagram as an SVG file. The bottom section allows entry or upload of a custom area specification, as well as offering controls to set the diagram size, colour palette and label sizes

The Edeap diagram generation tool (shown in Fig. 3) is available to use on the web at [20]. The source code is available from [22]. The open source implementation of Edeap allows users to integrate the software into their own systems, change parameters and modify the optimizer to their own preferences. The Edeap architecture and search mechanisms have been designed to be relatively simple, in part to allow for such modifications. As Edeap is a multi-criteria optimization system, users can add their own measures into the optimizer (for example to emphasize aesthetics they regard as important). This further differentiates Edeap from eulerr and venneuler, which only optimize a single criteria, stress, and so lack potential for such additions.

### Edeap optimiser

Edeap uses a hill climbing method to optimise a multi-criteria weighted sum objective function. Edeap currently uses the following criteria (described in the “Objective Function” section below), though more could be added.

- **RegionAreaDifference** (${\varvec{C}}_{1}$) A measure of the difference between the actual and desired area for each region.
- **missingOneLabelRegion** (${\varvec{C}}_{2}$) A measure of the amount that ellipses need to be moved to produce each missing (i.e., desired but not present) one-label region.
- **missingTwoOrMoreLabelRegion** (${\varvec{C}}_{3}$) A measure of the distance that pairs of ellipses need to be moved to produce components of any missing regions.
- **unwantedRegion** (${\varvec{C}}_{4}$) A measure of how far pairs of ellipses need to be moved apart to get rid of present but unwanted regions.
- **unwantedExpandedOverlap** (${\varvec{C}}_{5}$) A measure of how far each pair of ellipses that should not overlap need to be moved to produce a small separation between their boundaries.

All these criteria contribute in the objective function which is computed as follows:

$$\begin{aligned} \sum _{i=1}^{5} W_{i} C_{i} \end{aligned}$$

where $\textit{W}_{i}$ and $\textit{C}_{i}$ are the weight and the measure for criterion *i* respectively. Note that the value of each criterion is normalized so that it is between 0 and 1.

#### Neighbourhood search procedure

The Edeap optimiser begins with the necessary number of ellipses with the appropriate area and equal radius values (to initially configure them as circles), all positioned at (0, 0). For each ellipse, we apply systematic moves for exploring a neighbourhood solution:

1. Move the centre point of the ellipse in four directions (up, down, left, right) using a predefined distance (centerShift);
2. Increase/decrease the length of the major/minor axis of the ellipse using a predefined value (radiusLength);
3. Rotate the ellipse in both directions (clockwise/anticlockwise) using a predefined rotation angle (angle).

The hill climbing approach is described below in Algorithm 1. The objective function calculation is described in a dedicated section below. 
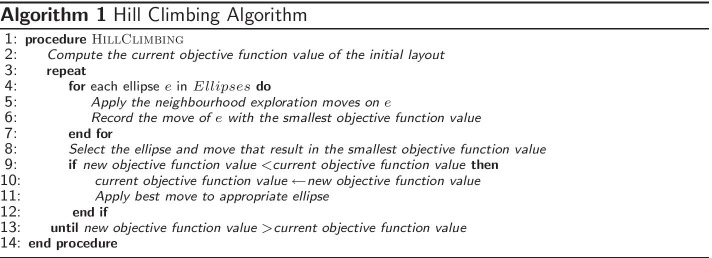


#### Equivalent ellipses

In the special case where the area specification contains multiple labels that are part of the exact same set of desired regions, we know that there are two (or more) ellipses that should precisely overlap. In this situation we treat these ellipses as having a single shared set of parameters, e.g., if we change the *x* value of one such ellipse we change it for all other equivalent ellipses. They are treated as individual ellipses for the purpose of objective function criteria calculations.

#### Weights and parameters tuning

We performed exploratory tests on a wide range of values for selecting proper initial values for each criterion’s weight and for the hill climbing algorithm’s parameters. Then we performed a systematic incremental procedure for tuning those values. This process for parameter tuning has been used in prior work [23, 24]. The process was divided into phases as follows:

1. In Phase I, we initially gave the same weight to all the criteria. Then, we picked a certain criterion, and we changed its weight to search for the value that gave the best result for the objective function. As the weight changed, we adjusted the other criteria weights to produce the same total sum of weights. Once the weight of the criterion was fixed, we moved to the next, until we had tuned the weights of all of the criteria.
2. In Phase II, we applied the same process performed in Phase I, starting with the weights from Phase I, but we chose the values that gave the minimum total area difference.

The tuning process was applied on diagrams with the properties listed in Table 2 (11 diagrams were generated from each group of diagrams):^1^

**Table 2:** Description of data used in weights and parameters tuning

Group	Ellipses	Intersections	Max intersection size
a	3	4	2
b	3	6	3
c	5	7	3
d	5	11	5
e	7	10	4
f	7	15	7

The random dataset generator we developed was used in the tuning process, as well as for comparing hill climbing and simulated annealing (see Hill Climbing vs. Simulated Annealing). It randomly produces intersections between 1 set and up to the “max intersection size” by picking a number between the limits and then selecting the required number of labels at random. It then assigns each set intersection a cardinality between 1 and 10. This is a relatively simple generator, and other models are possible. We note the analogy with random graph generation, which has been studied extensively [25]. However no such literature exists for Euler diagrams and an extensive investigation into random data generators for Euler diagram layout is beyond the scope of this paper.

After tuning the weights, the final selected values for the weights of the criteria in the objective function are listed below. The criteria weightings are those used in the online software and were used in all experiments and examples in this paper:

regionAreaDifference weight $$(\textit{W}_{1}) = 16.35$$

missingOneLabelRegion weight $$(\textit{W}_{2}) = 23.6$$

missingTwoOrMoreLabelRegion weight $$(\textit{W}_{3}) = 6.35$$

unwantedRegion weight $$(\textit{W}_{4}) = 0.01$$

unwantedExpandedOverlap weight $$(\textit{W}_{5}) = 3.6$$

and the values of the parameters of our optimiser are:

centerShift = 0.13

radiusLength = 0.03

angle = 0.1

#### Hill climbing versus simulated annealing

Hill Climbing (HC) is a fast neighbourhood search method. However, since it only takes steps in the direction of an optimal value, it can suffer from getting trapped in local optima within the search space. Simulated Annealing (SA), on the other hand, is a stochastic method that adds an element of non-determinism in order to escape from local optima. Simulated annealing is generally slower than hill climbing, but can lead to better results [26].

In order to test the effectiveness of our hill climbing algorithm, we compared it against simulated annealing. Before conducting the comparison, we had to tune the parameters of simulated annealing. The basic parameters include: initial temperature, cooling down rate, maximum number of iterations for running the algorithm, and number of iterations performed at each temperature.

We ran a similar tuning process to that for weights and hill climbing parameter tuning, see the Weights and Parameters Tuning Section, above. However, since simulated annealing is a stochastic method, we ran the simulated annealing procedure 10 times for each test case and took the mean of the results.

After determining the best values for simulated annealing parameters, we conducted a quick comparison against hill climbing by running both methods on new randomly generated datasets (described in Table 3).

**Table 3:** Description of data used in the comparison between hill climbing and simulated annealing

Group	Ellipses	Intersections	Max intersection size
a	3	3	2
b	3	7	3
c	5	6	4
d	5	12	6
e	7	9	4
f	7	14	6

From each group of diagrams listed in Table 3, we generated 10 test cases (60 diagrams total). We ran hill climbing once on all the test cases (since it is a deterministic method), then we computed the summation for each set of values of the four measures—area difference, objective function value, number of evaluated solutions, and execution time—for all the test cases in the same group. We then ran simulated annealing 10 times on each test case (stochastic method), we calculated the average of the 10 runs, then we computed the summation of averages for each set of values of the four measures for all the test cases that belong to the same group.

**Fig. 4:**
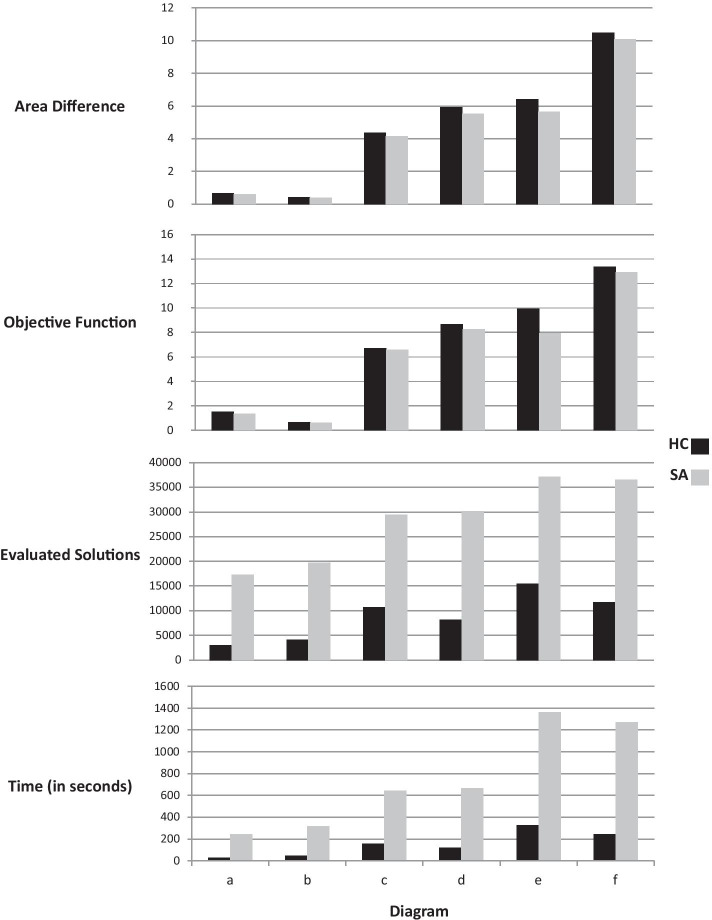
Hill Climbing (HC) versus Simulated Annealing (SA)

**Fig. 5:**
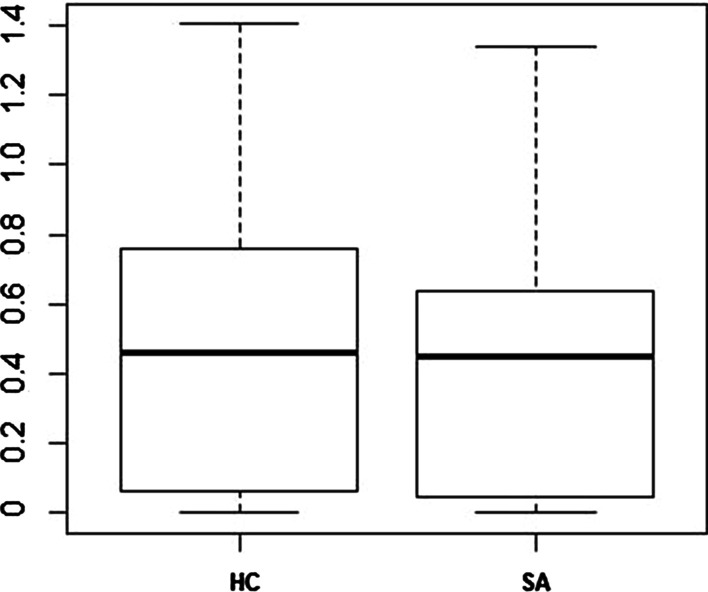
Area difference boxplot for Hill Climbing (HC) versus Simulated Annealing (SA)

Four bar charts are presented in Fig. 4 which describe the area difference (a box plot is also provided in Fig. 5), the objective function values, the number of evaluated solutions, and the execution time for both methods. We performed the non-parametric Wilcoxon rank sum test on area difference, objective function, number of evaluated solutions, and execution time with a confidence interval of 95% on the results of the 60 test cases (see Table 4). Also, in Table 4, we report the effect size to show how big the statistically significant differences are, using *Cohen d* measure [27]. This measures the sizes of differences between groups’ means in standard deviation units.

**Table 4:** p-values for Wilcoxon sum test with Bonferroni correction in NEJM format and Cohen’s d effect size interpretation for hill climbing versus simulated annealing run on Table 3 dataset

	Area difference	Objective function	Evaluated solutions	Time
p-values	0.71	0.63	< .001 (***)	< .001 (***)
Cohen d effect size	–	–	1 (large)	1.03 (large)

Following the New England Journal of Medicine (NEJM) practice [28], we regard p-values of less than 0.05 as statistically significant with one asterisk, p-values of less than 0.01 with two asterisks, and p-values less than 0.001 with three asterisks

There was no significant difference between simulated annealing and hill climbing for area differences and objective function values according to the results of Wilcoxon’s test, therefore, the effect size was not reported. On the other hand, with reference to Table 4 and Fig. 4, there is a statistically significant difference with large effect size in number of evaluated solutions and execution time attained by simulated annealing in comparison to hill climbing, which means that simulated annealing is slower (most of the cost of Edeap is in computing the objective function which is common to both techniques). Also, with simulated annealing, optimal solutions require the temperature to be decreased slowly. Otherwise, the resulting diagram is likely to be suboptimal. For these reasons, hill climbing was chosen as the preferred option to use in Edeap for the neighbourhood search algorithm.

### Edeap objective function

The objective function in Edeap consists of a number of weighted criteria, each described below.

#### Region area difference criterion

The region area difference criterion is calculated as the sum of differences between the desired proportion of total area for each region and the actual proportion of total area. This criterion represents the measure of how similar a generated diagram is to the given area specification. We use this measure for comparison of our results with other systems. It is analogous to the stress value used by venneuler and eulerr.

To compute the region area difference we first calculate the actual area for each region for the current ellipse parameters. We do this via point sampling because there is no current analytic solution to calculating areas of arbitrary numbers of overlapping ellipses (current methods for 3 ellipses rely on enumerating all possible overlap configurations [3], which is not feasible in the general case). We overlay a fixed-sized point grid over the diagram with a grid size of 0.026. To decide on this value, we tested a large range of grid sizes from 0.001 to 0.1 at 0.0002 increments, comparing them to the “accurate” reference grid size of 0.0002. A coarser discretisation will result in faster sampling but is less accurate, though the specific choice between grid steps makes only a small difference to the accuracy. We chose the largest grid size such that the difference from the reference grid size was less than 0.5% for all tested diagrams, and typically less than this. To ensure that the size of regions in the area specification do not affect accuracy when using the sampling approach, the initial parameters of all ellipses are scaled so that their area in the correct diagram would be 1.

In order to count the area of each region, we consider each intersection point in the overlaid grid. For each point we call a function for each ellipse to check whether the point lies inside that ellipse. As we do this, we keep the list of labels for the ellipses which that point lies within. We turn this list into a string which is used as the index into a dictionary that stores a count of pixels in each region. We also keep a count of the total number of pixels that lie inside at least one ellipse. From this info we can easily generate the proportion of the total area in each region. Since this criterion is generated using proportions of the total area, the absolute sizes of the areas given in the area specification do not matter, only their relative sizes.

While evaluating the many candidates at each iteration, only a single parameter of a single ellipse is modified for each candidate, while all other ellipses are left unchanged. In order to speed up our implementation, rather than call the function that determines if a point lies inside that ellipse, we keep a bitmap of each ellipse. For each point on the grid covered by the bounding-box of a given ellipse, this contains a boolean denoting whether the grid point is within the ellipse or not. During the frequent objective function calculations, this acts as a cache for the costly area calculations for the unchanged ellipses to save us from needlessly recomputing containment tests.

To calculate the value of our criterion we get the set of desired regions as well as the set of actual regions based on drawing a diagram with the current ellipse parameters. We iterate through each of the combined set of these regions. For each region that is both a desired (i.e., the set intersection is given in the area specification) and corresponding actual region (i.e., present given the combination of ellipses), we take the difference in their area proportions and add it to the criterion value. For each actual region without a corresponding desired region, we take the actual area proportion and add it to the criterion value. We subsequently refer to this as an *unwanted region*. Where a region is desired but there is no corresponding actual region, we take the desired area and add it to the criterion value. We refer to this as a *missing region*.

#### Unwanted region criterion

To compute this criterion, for each unwanted region $r_{unwanted}$, we check whether there is a desired region $r_{desired}$ that contains all the labels from region $r_{unwanted}$.

**Fig. 6:**
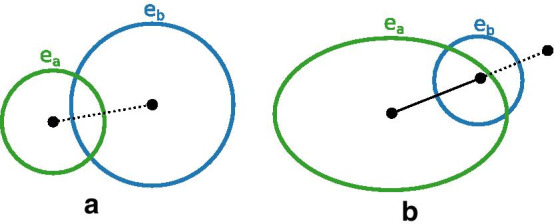
The unwanted region criterion. **a** Case 1 aims to remove unwanted region “a”, where there is a desired region “a b”. Ellipse $e_a$ should be moved to be positioned inside ellipse $e_b$ by penalising the area of region “a”. **b** Case 2 aims to remove unwanted region “a b” by inversely penalising the distance between of ellipses $e_a$ and $e_b$ while they overlap

There are two cases. The first is where there is a desired region $r_{desired}$ that contains all the labels from $r_{unwanted}$, and $r_{unwanted}$ has a single label. In this case, we add to the criterion the area of $r_{unwanted}$. The aim of this is to draw together an ellipse that should be positioned inside another ellipse, as with $e_a$ in Fig. 6a.

The second case is where there is no desired region that contains all the labels from $r_{unwanted}$. In this case, we attempt to separate pairs of ellipses in $r_{unwanted}$ that are not contained together in any desired region. To do this we consider each pair of labels in $r_{unwanted}$. If there is no desired region containing both those two labels, then we add to the criterion the ideal separation between the centre of the two corresponding ellipses minus the actual distance between their centres (as shown in Fig. 6b), where the ideal distance is the sum of the larger of each ellipses’ semi-minor and semi-major axis parameters. In order to break ties, for each matching pair beyond the first, we increase a factor applied to this distance by 0.1 (beginning with 1). The purpose of this is to push apart ellipses that form an unwanted region where there is no other desired region that includes the same pair of ellipses.

#### Missing one-label region criterion

The purpose of the missing one-label region criterion is to push ellipses away from all others where the ellipse corresponds to desired, but not present, one-label region, i.e., an ellipse that should not overlap any other ellipses.

To compute this criterion for each missing one-label region $r_{missing}$, we consider the same measure as for the unwanted region criterion, i.e., how much overlap there is between the corresponding ellipse $e_{missing}$ and other ellipses (see Fig. 6b). Hence, for each ellipse $e_{overlap}$ that overlaps with $e_{missing}$ we add to the criterion the ideal separation between the centres of $e_{missing}$ and $e_{overlap}$ minus the actual distance between their centres, where the ideal distance is the sum of the larger of each ellipses’ semi-minor and semi-major axis parameters.

#### Missing two-or-more-label region criterion

The purpose of the missing two-or-more-label region criterion is to pull individual ellipses towards groups of overlapping ellipses when all these ellipses have the labels in a desired but not present region.

To compute this criterion, for each missing region $r_{missing}$ containing two-or-more labels we select the actual region $r_{common}$ that shares the most labels with $r_{missing}$. In the case of a tie for regions that share the most labels, we pick the region with the overall fewest number of labels. Once we have selected region $r_{common}$ there are two possible cases. The region $r_{common}$ might not contain all the desired labels from the missing region $r_{missing}$. It is also possible that the region $r_{common}$ contains all the labels of $r_{missing}$ and some other labels too. We treat these cases differently.

**Fig. 7:**
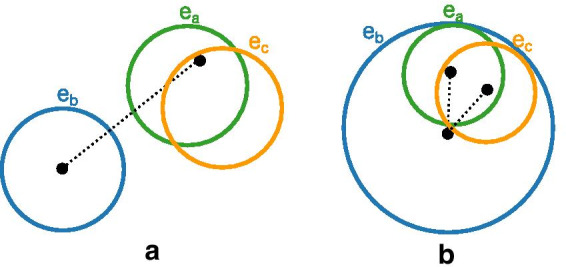
Missing two-or-more-label region criterion. **a** Case 1 draws the ellipse $e_b$ towards the region “a c” by penalising the distance between the centre of $e_b$ and a known point in region “a c”. **b** Case 2 aims to push the ellipse $e_b$ away from both ellipses $e_a$ and $e_c$ to create the missing region “a c”. Once region “a c” exists the region area difference criterion will work to adjust to the appropriate region proportions, but can’t do this until the region “a c” is created (outside of “a b c”)

If the region $r_{common}$ does not contain all the desired labels from the $r_{missing}$, we find the set of labels in $r_{missing}$ that are not in $r_{common}$. Ellipses with these labels are ones we wish to move towards $r_{common}$ to to produce our desired region. For each of these labels we add to the criterion the difference between the centre of the ellipse and a reference point known to be inside $r_{common}$ (as shown in Fig. 7a).

If the region $r_{common}$ contains all the labels of $r_{missing}$ and other (undesired) labels too, we wish to push “extraneous” ellipses with these unwanted labels away from region $r_{common}$. We aim to separate each extraneous ellipse from each of the ellipses with a label in region $r_{missing}$ by adding to the criterion the ideal separation between the centre of each pair of these ellipses minus the actual distance between their centres, where the ideal distance is the sum of the larger of each ellipses’ semi-minor and semi-major axis parameters (as shown in Fig. 7b).

#### Unwanted expanded overlap criterion

The *region area difference criterion* already penalises overlap between pairs of ellipses which are known not to exist together in any desired region, but does nothing to move such ellipses when they no longer overlap, even if their boundaries touch. Ideally, such ellipses would be positioned slightly apart, rather than touching each other. The *unwanted expanded overlap criterion* penalises such closeness by simply expanding the boundaries of these ellipse pairs and adding to the criterion value the sum of all overlap between these expanded ellipses. The semi-minor and semi-major axis parameters of each of these ellipses are expanded by 15% of the larger of that ellipse’s axis parameters. This criterion does nothing to change the correctness of the diagram (which is equally correct if such ellipses touch or don’t), but does improve the readability and thus usability of the generated diagrams.

### Edeap label placement

**Fig. 8:**
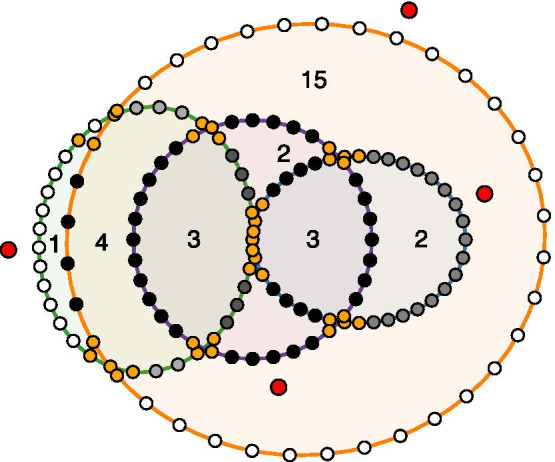
Dots showing positions considered when determining candidate ranges (lighter consecutive dots) for final ellipse label positions (red dots) that are away from ambiguous locations (orange dots)

We want ellipse labels (coloured labels) to be positioned as unambiguously as possible. To compute label positions in Edeap, we check 36 *boundary points* (at 10 degree angles) around the periphery of each ellipse. We assign each a *depth* value equal to the number of other ellipses it lies within and a *distance* value equal to the closest boundary point from any other ellipse. This depth is indicated by the darkness of each point in Fig. 8 (white points lie outside all other ellipses). We partition these points into ranges of consecutive points with the same depth, further partitioning these at “ambiguous” points whose distance value is within a certain threshold (points marked orange in Fig. 8). The label for each ellipse is positioned on that ellipse’s longest range with the lowest depth, at that range’s point with the highest distance value (i.e., as far from other ellipse boundary points as possible).

To determine positions for each region label (black labels showing numbers), Edeap keeps track of the first point $p_{first}$ encountered in any region for each unique combination of labels. It also keeps track of an average position $p_{average}$ for all points found to be in this region. It determines a potential viable label position $p_{viable}$ by checking if $p_{average}$ is inside the same region (it may not be; such as in the case of a split region). If it is, we choose a viable point $p_{viable} = p_{average}$, or if not, $p_{viable} = p_{first}$. Edeap then scans vertically from that *x* position of $p_{viable}$ to find the vertical bounds of that region, then scans horizontally from the *y* position of $p_{viable}$ to find the horizontal bounds. The position of $p_{viable}$ is updated to be the centre of the horizontal and vertical bounds. This scanning and centering process is repeated several times.

### Other optimisation methods

The current method explored only hill climbing and simulated annealing. It might be possible to get better results using improved optimization methods that have shown good results with multi-criteria graph layout, such as Tabu Search and Path Relinking [26]. Tabu search uses a memory list to speed up the searching process by avoiding previously visited solutions while path relinking generates new solutions by exploring paths that connect high quality solutions selected from an elite set of solutions. Other neighbourhood search-based methods can be considered such as Variable Neighbourhood Search and Extremal Optimization. Population-based optimization methods including Genetic Algorithms, Particle Swarm Optimization, Social Cognitive Optimization, and Ant Colony Optimization are also potential candidates for experimentation [29], possibly improving the final result, though at the cost of increased run time. It would also be possible to explore different starting diagrams, including using the output of the fast venneuler system, although this would require a re-implementation of the venneuler code to work with our client-side JavaScript implementation.

## Results

We conducted a performance and quality comparison of the three area-proportional Euler diagram drawing methods, Edeap, eulerr and venneuler, by running them on real world data from two sources. Firstly, we accessed Twitter data from the SNAP data set [30] in the form of a number of Twitter circles, or interest groups. This gave us an unbiased test set that represented data that users might wish to visualize. Secondly, we utilised data from the Gene Ontology (GO) database [31] accessed through VennMaster [2]. We also tested all three systems for scalability on a randomly generated dataset. This work is the only comparison that we are aware of for general area-proportional Euler diagram generation with different shapes.

Given the variation in ways of measuring accuracy (Edeap uses area difference whereas venneuler and eulerr use stress as a target function), we report results for both measures. We also evaluated the time to reach a solution on the same hardware. As venneuler and eulerr are stochastic methods, we ran the tests 10 times. We used the default parameters for all three software systems. The experiments were conducted on a 2.9 GHz Intel Core i7 MacBook Pro with 16GB RAM running macOS Catalina version 10.15.6. We used venneuler version 1.1-1 and eulerr version 6.1.0. Edeap was run in Electron 2.0.3. No other applications were running during the tests. We set a time-out of 30 minutes for each system on each test.

All test data, a summary of the characteristics of the data, and results including diagrams generated by each system for all tests are available to browse [32] or download [33].

To ensure a fair comparison, we took the ellipse parameters output by venneuler and eulerr and used the Edeap code to position labels, render the final image, and to calculate both stress and area difference values for all three systems.

In each experiment, we applied the Wilcoxon signed-rank test with Bonferroni correction (pairwise comparison) with a confidence level of 95% to see how significant the difference was between pairs of methods. We chose Wilcoxon signed-rank test [34] after applying Shapiro-Wilk’s normality test [35] on the data with a 95% confidence level. The p-value of Shapiro-Wilk’s test was below 0.05. Thus, the null hypothesis of Shapiro-Wilk’s test that the population is normally distributed was rejected. We also followed the guidelines suggested by Cohen [27] and Sawilowsky [36] for interpretation of the effect size using Cohen’s *d* measure that indicates the standardised difference between two means.

### Twitter SNAP dataset

This data set included 774 area specifications derived from the SNAP data set [30], in particular the Twitter social circles data, which gives user interests. Each social circle file consists of information about which users have which interests. Each file formed an area specification. The interests were the sets, users sharing collections of interests were the set intersections, and the number of users sharing the collections of interests were the cardinalities of the intersections. All data was relabelled.

**Fig. 9:**
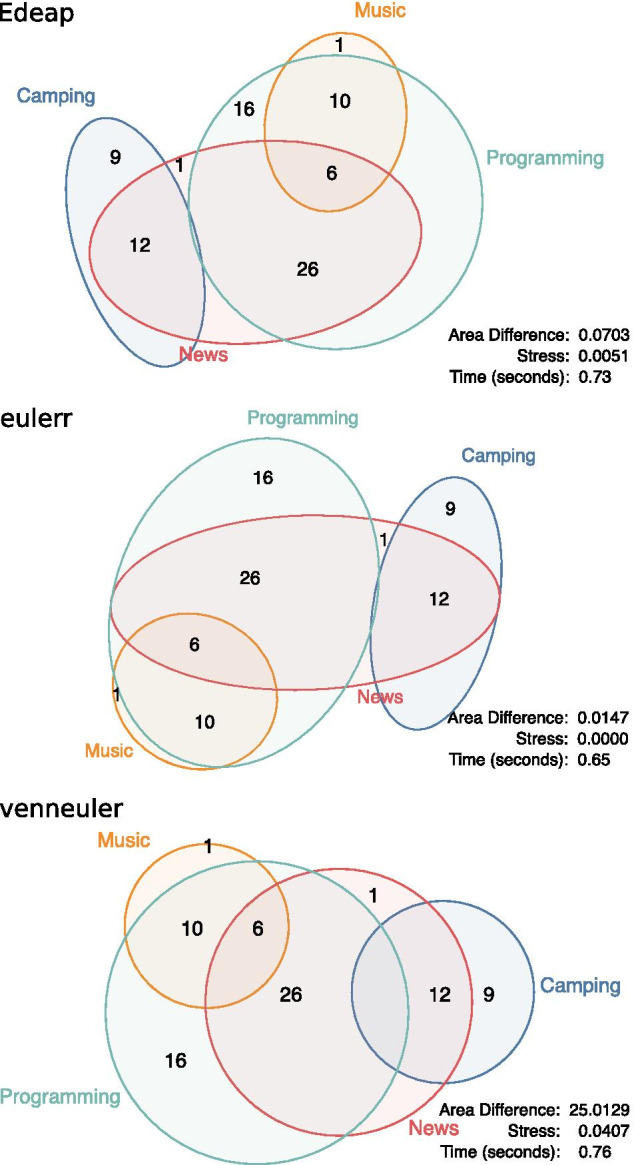
Example output of an area specification produced from the SNAP data set

An example area specification from the SNAP data set is given below. Figure 9 shows the resultant diagrams from the first run.

Programming News 26         News 1

Programming News Music 6    Music 1

Programming Music 10        Camping 9

Camping News 12             Programming 16

**Fig. 10:**
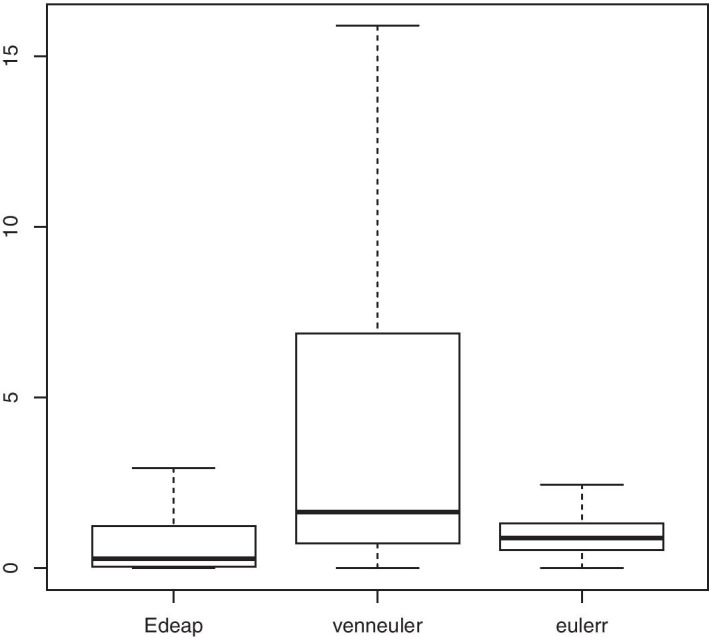
Boxplot (excluding maximum outliers) for area difference on SNAP dataset

**Fig. 11:**
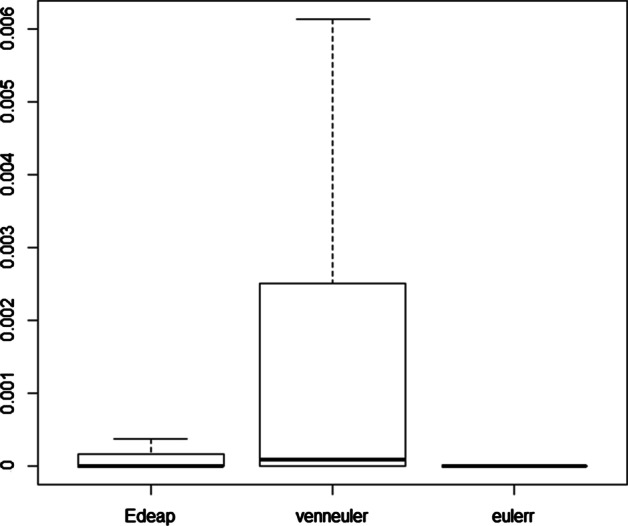
Boxplot (excluding maximum outliers) for stress on SNAP dataset

**Fig. 12:**
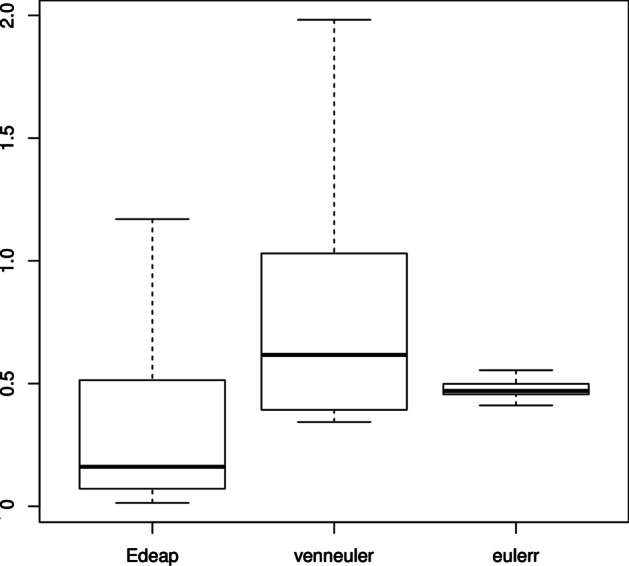
Boxplot (excluding maximum outliers) for time (in seconds) on SNAP dataset

Figures 10, 11, and 12 show boxplots for the measures of area difference, stress, and time respectively on SNAP dataset. Whereas Tables 5 and 6 show the p-values for Wilcoxon signed-rank test with Bonferroni correction, and Cohen’s *d* measure for effect size respectively. As we mentioned earlier, with respect to NEJM practice [28], we regard p-values of less than 0.05 as statistically significant with one asterisk, p-values of less than 0.01 with two asterisks, and p-values less than 0.001 with three asterisks. Note that, 12 diagrams from the SNAP dataset, that contain at least 12 ellipses, were excluded as eulerr timed-out and failed to generate layouts. Furthermore, in the boxplots, outliers were far away from the median and the 3rd quartile. Therefore, we removed the maximum outliers to get a clear distribution of the data in the boxplot.

**Table 5:** p-values for Wilcoxon signed-rank test with Bonferroni correction on SNAP dataset

	Area difference	Stress	Time
Edeap versus venneuler	< .001 (***)	0.031 (*)	< .001 (***)
Edeap versus eulerr	0.024 (*)	0.033 (*)	< .001 (***)
venneuler versus eulerr	< .001 (***)	<.001 (***)	< .001 (***)

Following the New England Journal of Medicine (NEJM) practice [28], we regard p-values of less than 0.05 as statistically significant with one asterisk, p-values of less than 0.01 with two asterisks, and p-values less than 0.001 with three asterisks

**Table 6:** Cohen’s *d* effect size interpretations for SNAP dataset

	Area difference	Stress	Time
Edeap versus venneuler	0.8 (large)	0.01 (very small)	0.1 (small)
Edeap versus eulerr	0.33 (moderate)	0.01 (very small)	14.5 (very large)
venneuler versus eulerr	0.85 (large)	0.01 (very small)	14.3 (very large)

We can see from Fig. 10 and area difference column in Tables 5 and 6 that, regarding area difference, there are significant differences between all methods with large effect size in favour of Edeap over venneuler, moderate effect size for eulerr over Edeap, and large effect size for eulerr over venneuler. Note that the maximum area difference values were 125.75 for Edeap, 200.00 for venneuler, and 91.24 for eulerr.

We can see from Fig. 11 and stress column in Tables 5 and 6 that, regarding stress, there are again significant differences between all methods but with very small effect size which means that the difference is trivial. Note that the maximum stress values were 0.76 for Edeap, 1.00 for venneuler, and 0.40 for eulerr.

We can see from Fig. 12 and time column in Tables 5 and 6 that, regarding time, there are significant differences between all methods with small effect size in favour of venneuler over Edeap, but huge effect size for both over eulerr. Note that the maximum values of time in seconds were 19.12 for Edeap, 4.47 for venneuler, and 1220.0 for eulerr.

### Gene ontology dataset

The Gene Ontology dataset was derived from the GO database [31]. As in the VennMaster paper [2] the overlap of genes in different GO categories results from the association of genes with multiple GO categories. Two filtering mechanisms are used, a p-value limit and a range of interest for the number of genes in a particular category. To produce a suitable variety of data sets, we produced data sets for all combinations where the p-value went from 0.01 to 0.09 in increments of 0.01, the minimum interest range went from 10 to 90 in increments of 5, the maximum interest range went from 100 to 180 in increments of 10. This resulted in 124 area specifications. It is worth noting that 24 diagrams, that contain at least 12 ellipses, were excluded as eulerr timed-out and failed to generate layouts.

**Table 7:** p-values for Wilcoxon signed-rank test with Bonferroni correction on Gene Ontology dataset

	Area difference	Stress	Time
Edeap versus venneuler	< .001 (***)	< .001 (***)	< .001 (***)
Edeap versus eulerr	< .001 (***)	< .001 (***)	0.002 (**)
venneuler versus eulerr	< .001 (***)	< .001 (***)	0.004 (**)

Following the New England Journal of Medicine (NEJM) practice [28], we regard p-values of less than 0.05 as statistically significant with one asterisk, p-values of less than 0.01 with two asterisks, and p-values less than 0.001 with three asterisks

**Table 8:** Cohen’s *d* effect size interpretations for Gene Ontology dataset

	Area difference	Stress	Time
Edeap versus venneuler	0.4 (moderate)	0.4 (moderate)	1 (large)
Edeap versus eulerr	0.16 (small)	0.18 (small)	2.6 (very large)
venneuler versus eulerr	0.59 (moderate)	0.56 (moderate)	3.6 (very large)

**Fig. 13:**
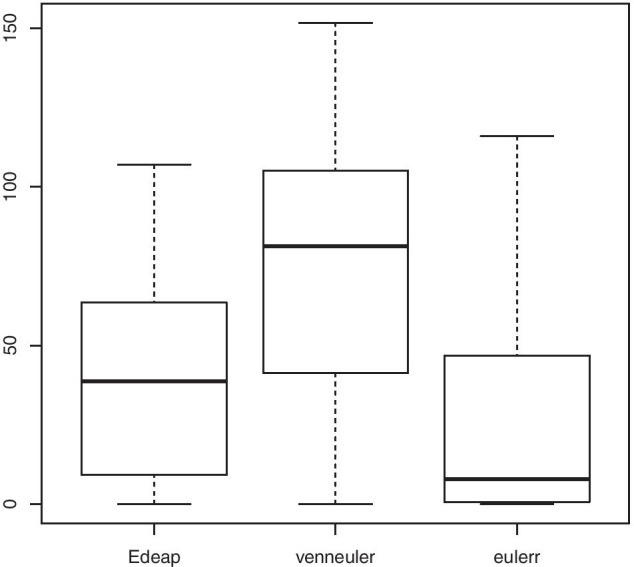
Boxplot for area difference on Gene Ontology dataset

We can see from Fig. 13 and area difference column in Tables 7 and 8 that, regarding area difference, there are significant differences between all methods with moderate effect size in favour of Edeap over venneuler, small effect size for eulerr over Edeap, and moderate effect size for eulerr over venneuler.

**Fig. 14:**
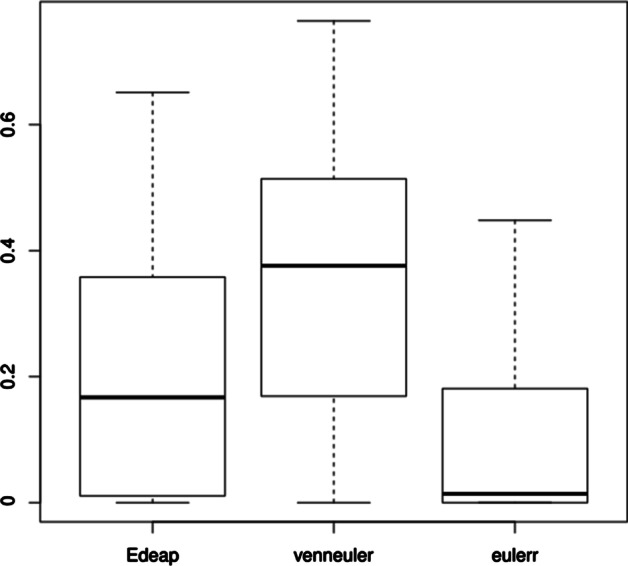
Boxplot for stress on Gene Ontology dataset

Regarding the stress, we see from Fig. 14 and stress column in Tables 7 and 8 that there are also significant differences between all methods with small effect size for eulerr over Edeap, and moderate effect size in favour of both over venneuler.

**Fig. 15:**
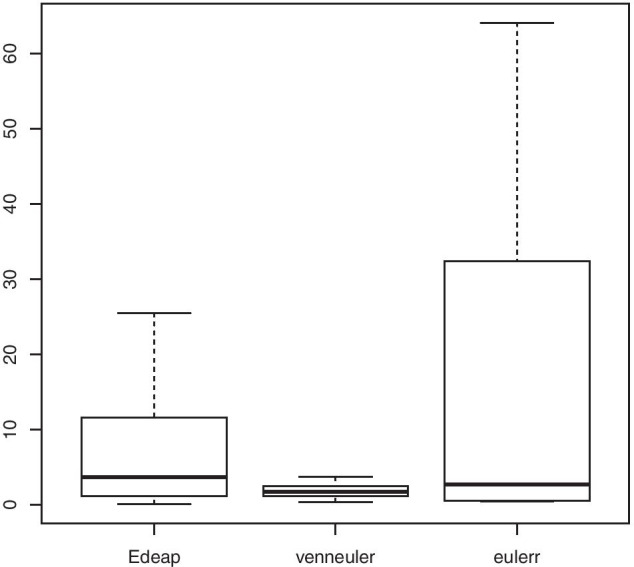
Boxplot (excluding maximum outliers) for time (in seconds) on Gene Ontology dataset

As for execution time, clear differences between the three methods can be recognized in Fig. 15 and time column in Tables 7 and 8 with large effect size in favour of venneuler over Edeap, but with very large effect size for both over eulerr. Note, outliers were far away from the median and the 3rd quartile in eulerr boxplot and were thus removed in the boxplots to see a clearer distribution of the data.

### Scalability experiment

To investigate the scalability of the methods, we ran each method on 90 randomly generated diagrams produced by the following method:

1. first, we randomly generated a number of sets, *s*, between 3 and 20;
2. For each *s* we randomly generated a set of 5 diagrams such that number of intersections was each of {s*1, s*1.5, s*2, s*2.5, s*3};
3. for each intersection in each diagram, the number of sets was randomly generated to be between 1 and 4;
4. for each intersection, we randomly generated the cardinality to be an integer between 1 and 10.

**Fig. 16:**
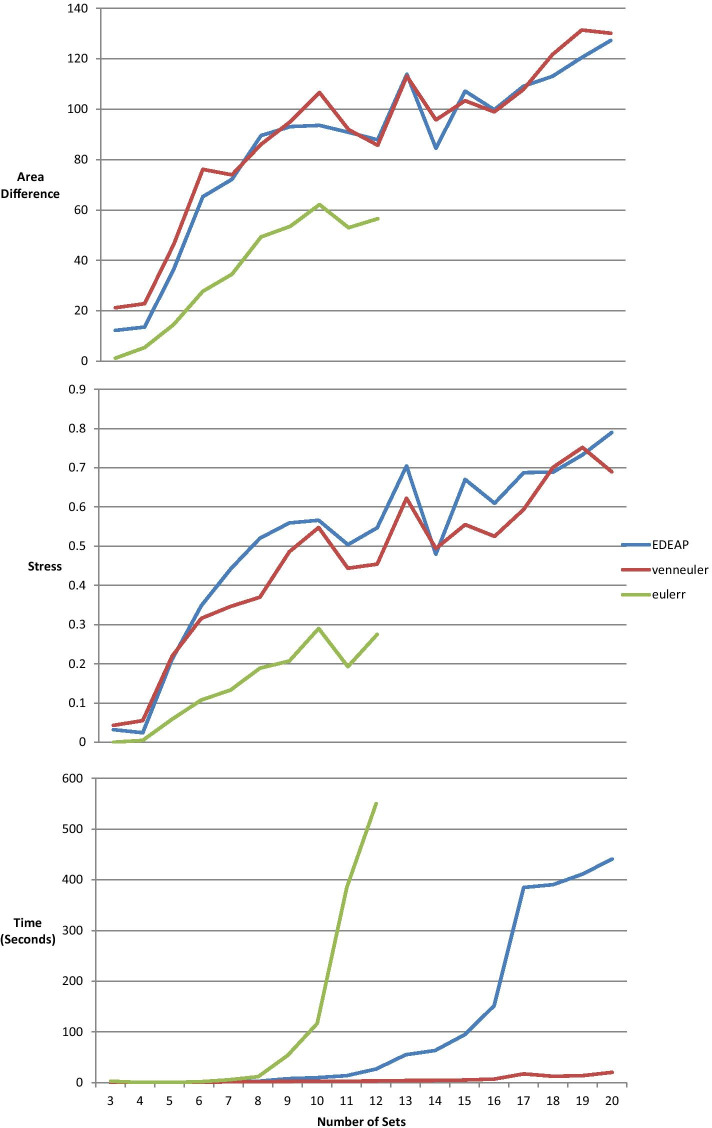
The effect of increasing number of sets in the area specification on area difference, stress, and execution time, for the three systems

Figure 16 shows the effect of increasing the size of the diagram on area difference, stress, and execution time. The eulerr data is not complete because, except for trivial cases (such as diagrams drawable with disconnected circles), the system timed-out for data beyond 11 sets.

## Discussion

When evaluating systems, we take the standard approach of ordering by importance, (1) production of a visualization (2) accuracy of the representation and (3) time taken to generate the visualization.

**Fig. 17:**
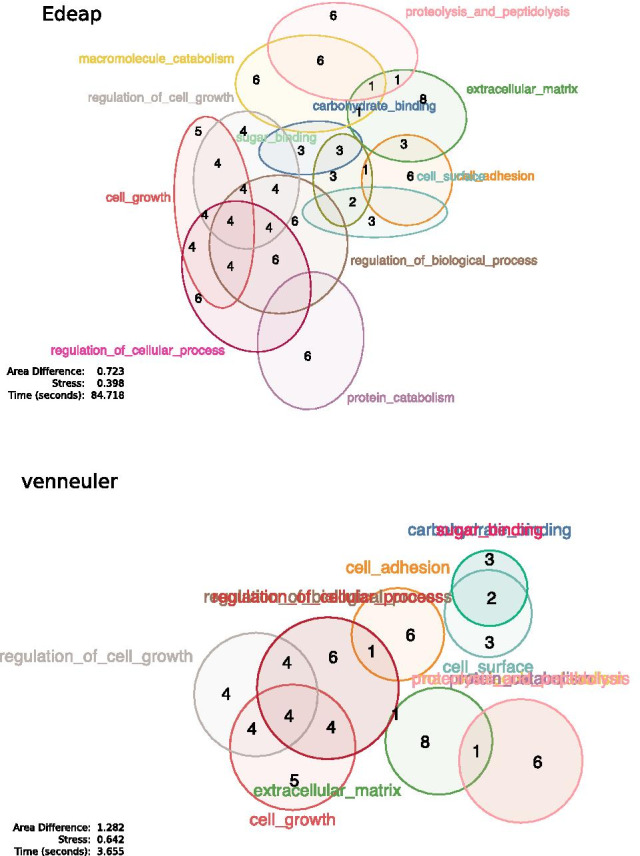
Scalability example, drawable with Edeap and VennMaster, not drawable with eulerr. GO Miner filters, p-value: 0.03, minimum: 20, maximum 160

On these measures, beyond 11 sets, eulerr fails due to inability to generate a visual representation. Edeap is more accurate than venneuler, hence we regard Edeap as the most scalable method for drawing area-proportional Euler diagrams with ellipses. An example of a 12 set diagram is shown in Fig. 17, drawn with Edeap and venneuler, but which is not drawable with eulerr. Its area specification is given by the file figure17areaSpecification.txt in [33]. We conjecture that the problems that eulerr has with drawability at this scale is due the large inaccuracies in diagrams at this size. Hence its slow last-ditch method is applied for nearly all data sets of this size.

At set sizes between 2 and 11, eulerr is significantly more accurate in representing area specifications than Edeap, which in turn is significantly more accurate than venneuler. There was significance in both area difference and stress. We note that eulerr and venneuler had better results when measuring stress, however, this is to be expected as these two systems aim to minimize stress, whereas Edeap does not. We conclude that at smaller set sizes, for highest accuracy, eulerr is the preferred technique.

The timing is reversed, with venneuler being the fastest, followed by Edeap, with eulerr being the slowest. Effect sizes are particularly large for time, and if time performance is critical then venneuler would be the preferred choice.

## Conclusions

We have developed a web-based open source software system, Edeap, for the automatic drawing of any area-proportional Euler diagrams with ellipses.

We compared Edeap with two competing methods, venneuler and eulerr. eulerr produces more accurate results at smaller diagram size, but in our required timeframe it is unable to generate results at larger diagram size, where the most accurate results are produced by Edeap. venneuler is far faster than the other two methods, but less accurate. Hence we have the following advice when selecting a system:

- for data with more than 11 sets, Edeap is the preferred method
- for data with 11 sets or fewer, eulerr is the preferred method
- if time is the critical constraint, venneuler is the preferred method

Edeap’s multi-criteria approach means it is possible to add additional criteria to increase accuracy, as well as optimizing on other features of the layout that improve readability (possibly at the cost of accuracy, as these may complete). For example, the *unwanted expanded overlap criterion* does not help separate ellipse boundaries where one ellipse is inside another, i.e., the inner ellipse bumping into the boundary of the outer ellipse. It would be possible to penalize this by reducing the size of the outer ellipse and penalizing any area of the inner ellipse that is outside of the reduced-size outer ellipse. Another possible addition is to add a circle-distortion criterion that adds a slight penalty each ellipse based on the difference between each ellipse’s semi-minor and semi-major axis parameters, i.e., how far it is away from being a circle. Our current region area difference penalises a region area that is 49 instead of 50 as much as a region that is 1 instead of 2 (when they are in the same instance). The difference to the larger region may matter less to the reader, hence it could be worth investigating how to consider the difference to the smaller region as more important in our objective function.

Current research has restricted the use of shapes to circles, ellipses and rectilinear curves. Future work might consider more generalised ovaloid shapes in place of ellipses, which might potentially making many more area specifications drawable. However, as the shapes become more complex, it may be that the diagrams are made less usable.

### Availability and requirements

**Project name:** Edeap

**Project home page:** https://www.eulerdiagrams.com/edeap/

**Archived version:** https://doi.org/10.26180/13168154

**Operating system(s):** Platform independent

**Programming language:** JavaScript

**Other requirements:** Google Chrome web browser

**License:** GNU General Public License v3.0

**Any restrictions to use by non-academics:** None

## Data Availability

The dataset(s) supporting the conclusions of this article are available in the Monash University figshare repository, https://doi.org/10.26180/13168121.

## Footnotes

1 Experimentation of the tuning process showed that the order of tuning the values of weights and criteria in Phase II had an impact on the final results and so we used an order that produced, on average, the best results.
